# Supplementary material for: Stochastic pulsing of gene expression enables the generation of spatial patterns in Bacillus subtilis biofilms
Source: Nat Commun. 2020 Feb 19;11:950. doi: 10.1038/s41467-020-14431-9 (PMC7031267; doi:10.1038/s41467-020-14431-9)
Supplement: Supplementary file 1 — Supplementary Information [file 41467_2020_14431_MOESM1_ESM.pdf]

Stochastic pulsing of gene expression enables the generation of spatial patterns in *Bacillus subtilis* biofilms

Eugene Nadezhdin[1,†], Niall Murphy[1,2,†],
Neil Dalchau[2], Andrew Phillips[2], and James C.W. Locke[1,2,3,*]

[1]Sainsbury Laboratory, University of Cambridge, Cambridge CB2 1LR, UK
[2]Microsoft Research, 21 Station Rd, Cambridge CB1 2FB, UK
[3]Department of Biochemistry, University of Cambridge, Cambridge CB2 1QW, UK
[†]These authors contributed equally to this work.
[*]Correspondence should be addressed to E-mail: james.locke@slcu.cam.ac.uk

1 Supplementary Information

1.1 Supplementary Methods

1.1.1 Plasmid construction

Plasmids were cloned using *E. coli* strain DH5α and regular cloning methods. Plasmid constructs were integrated into the *B. subtilis* chromosome via double crossover using standard techniques. The following list provides a description of the plasmid constructed, with details on integration position/cassette and selection marker given at the beginning.

1.1.2 Promoter definitions

1. P_{sigA}– The σ^A binding site, (-47 to +18 from the transcriptional start site) of the promoter from the *trpE* gene, as used in [1, 2].

2. P_{sigB}– The promoter immediately upstream of *rsbV*, containing a well-characterized σ^B promoter [1].

3. P_{spoIID}– the 232bp upstream of the *spoIID* start codon as used in [2].

4. P_{sspB} – The 250bp upstream of the *sspb* start codon as used in [3].

1.1.3 Plasmid list

1. sacA::P_{sspB}-yfp (cm) – The late sporulation gene *sspB* promoter was amplified by PCR using the pair of primers P_sspB_EcoRI_F: CCGGAATTCCAAACGAGATACATGAACTGATG and P_sspB_BamHI_R: GGCGGATCCCTTTTTATTTAGTATGGTTGGG (modified from [3] and NCIB3610 genomic DNA as a template with Phusion HF DNA polymerase (NEB). PCR fragment was purified using standard techniques, digested with EcoRI and BamHI and cloned into EcoRI/BamHI sites of plasmid AEC127 (Gift of Dr. A. Eldar [2]), yielding YFP (Venus) reporter under

control of an *sspB* promoter. The plasmid replicates in *E. coli* but not in *B. subtilis*.

2. Plasmid ECE174-P$_{ydbS}$-YFP from [4].

The *B. subtilis* strain 168 Marburg was the genetic background of the strains used as donors of genomic DNA. NCIB3610 was used as the background for biofilm experiments. Deletions were made by replacing genes of interest with a selection marker via a linear DNA fragment homologous to the region of interest. Please refer to Supplementary Table 1 for description of strains and their construction. Under the construction procedure, the "→" symbol indicates an integration event from plasmid or genomic DNA fragment into the strain after the arrow. For example, in strain JLB021 the construction procedure is listed as "JJB213→JLB035 (with cm selection)", meaning "the genomic DNA of JJB213 was prepared and transformed into JLB035, with selection on cm".

Strain	Genotype	Construction	Reference/source
NCIB3610	Wild type, undomesticated biofilm capable strain		BGSC 3A1
3A38	WT, *comIQ12L*		BGSC 3A38 [5]
BKK23450	*trpC2 sigF*::*kan*		BGSC BKK23450 [6]
JJB153	*trpC2 ppsb*::P_{trpE}-*mCherry* (kan)		Gift of M. Elowitz
JJB155	*trpC2 ppsb*::P_{trpE}-*mCherry* (phleo)		Gift of M. Elowitz
JJB213	*trpC2 ppsb*::P_{trpE}-*mCherry* (phleo) *sacA*::P_{sigB}-*yfp* (cm)		[1]
JJB417	*trpC2 ppsb*::P_{trpE}-*mCherry* (phleo) *rsbR-rsbU*::*erm amyE*::*rsbQ-rsbP* (spc)		Gift of M. Elowitz
JJB556	*trpC2 ppsB*::P_{trpE}-*mCherry* (neo) Δ*rsbU*; *amyE*::P_{sigB}-3×*cfp* (spec) *sacA*::P_{yflA}-*yfp* (cm)		Gift of M. Elowitz
JJB559	*trpC2 ppsB*::P_{trpE}-*mCherry* (neo) Δ*rsbU*; *amyE*::P_{sigB}-3×*cfp* (spec) *sacA*::P_{csbB}-*yfp* (cm)		Gift of M. Elowitz
JJB635	JJB213; *rsbR-rsbU*::*erm*		[1]
JJB637	JJB213; *rsbR-rsbU*::*erm rsbQP*::*tet*		[1]
PB344	*trpC2 sigB* Δ3::*spc*		BGSCID 1A780
AES243	*trpC2 amyE*::P_{spoIID}-*cfp* (spc)		Gift of A. Eldar
AES1449	*trpC2 ppsb*::P_{trpE}-*mCherry* (phleo) *sacA*::P_{trpE}-*yfp* (cm)		Gift of A. Eldar
JLB021	*ppsb*::P_{trpE}-*mCherry* (kan) *sacA*::P_{sigB}-*yfp* (cm)	JJB213→JLB035 (with cm selection)	This study
JLB022	*ppsb*::P_{trpE}-*mCherry* (kan) *sacA*::P_{ydbS}-*yfp* (cm)	ECE174-P_{ydbS}-*yfp*→JLB035 (with cm slection)	This study
JLB035	*ppsb*::P_{trpE}-*mCherry* (kan)	JJB153→NCIB3610 (with kan selection)	This study
JLB039	*ppsb*::P_{trpE}-*mCherry* (kan) *sacA*::P_{sigB}-*yfp* (cm) rsbQP::tet	JJB637→JLB021 (with tet selection)	This study
JLB077	*ppsb*::P_{trpE}-*mCherry* (kan) *sacA*::P_{sspB}-*yfp* (cm)	JLB079→JLB035 (with cm selection)	This study
JLB079	*trpC2 ppsb*::P_{trpE}-*mCherry* (phleo) *sacA*::P_{sspB}-*yfp* (cm)	plasmid (1, from plasmid list) → JJB155 (with cm selection)	This study
JLB088	*ppsb*::P_{trpE}-*mCherry* (kan) *sacA*::P_{sigB}-*yfp* (cm) *rsbR-rsbU*::*erm*	JJB635→JLB021 (with erm selection)	This study
JLB094	*comIQ12L sacA*::P_{sigB}-*yfp* (cm)	JJB213→3A38 (with cm selection)	This study
JLB095	*ppsb*::P_{trpE}-*mCherry* (kan) *sacA*::P_{sigB}-*yfp* (cm) *amyE*::*rsbQ-rsbP* (spc)	JJB417→JLB021 (with spc selection)	This study
JLB098	*ppsb*::P_{trpE}-*mCherry* (kan) *sacA*::P_{sigB}-*yfp* (cm) *sigB* Δ3::*spc*	PB344→JLB021 (with spc selection)	This study
JLB104	*comIQ12L ppsb*::P_{trpE}-*mCherry* (phleo)	JJB155→3A38 (with phleo selection)	This study
JLB106	JLB094; *ppsb*::P_{trpE}-*mCherry* (phleo)	JJB155→JLB094 (with phleo selection)	This study
JLB117	*ppsb*::P_{trpE}-*mCherry* (kan) *sacA*::P_{sspB}-*yfp* (cm) *amyE*::*rsbQ-rsbP* (spc)	JJB417→JLB077 (with spc selection)	This study
JLB118	*ppsb*::P_{trpE}-*mCherry* (kan) *sacA*::P_{sspB}-*yfp* (cm) *sigB* Δ3::*spc*	PB344→JLB077 (with spc selection)	This study
JLB124	*ppsb*::P_{trpE}-*mCherry* (kan) *sacA*::P_{sigB}-*yfp* (cm) *amyE*::P_{spoIID}-*cfp* (spc)	AES243→JLB021 (with spc selection)	This study
NEB007	JLB104; *rsbR-rsbU*::*erm*	JLB088→JLB104 (with erm selection)	This study
NEB008, NEB009	JLB106; *rsbR-rsbU*::*erm*	JLB088→JLB106 (with erm selection)	This study
NEB011, NEB012	NEB008; *amyE*::*rsbQP* (spc)	JLB095→NEB008 (with spc selection)	This study
NEB018	NEB008; *sigF*::*kan*	BKK23450→NEB008 (with kan selection)	This study
NEB024, NEB025	NEB007; *sacA*::P_{yflA}-*yfp* (cm)	JJB556→NEB007 (with cm selection)	This study
NEB026, NEB027	NEB007; *sacA*::P_{csbB}-*yfp* (cm)	JJB559→NEB007 (with cm selection)	This study
NEB034	NEB007; *sacA*::P_{trpE}-*yfp* (cm)	AES1449→NEB007 (with cm selection)	This study

Supplementary Table 1. Strain information and construction. Antibiotics: spectinomycin (spc), kanamycin (kan), chloramphenicol (cm), tetracycline (tet), erythromycin (erm) and phleomycin (phleo).

1.2 Supplementary Figures

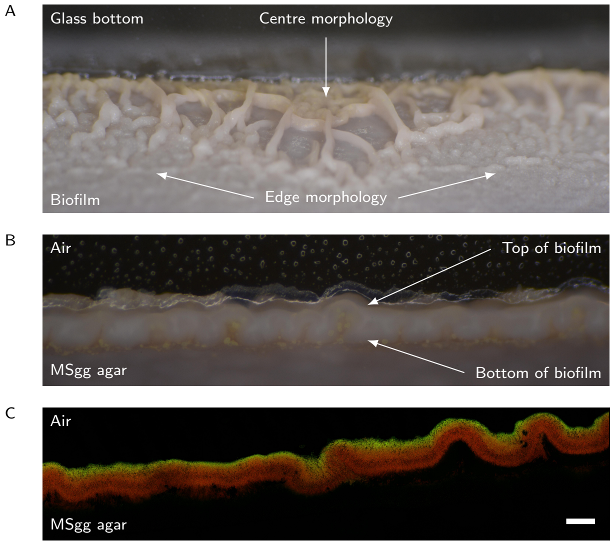

Supplementary Fig. 1. Biofilms grown against the bottom of a glass bottom dish display the same morphology as biofilms grown in standard conditions. **(A)** Image of biofilm growing on the MSgg agar surface at 96 hours. The typical wrinkle morphology is visible. The biofilm grows pressed up against the glass bottom which is visible at the top of the image. **(B)** Cross-sectional view of the biofilm through the glass bottom coverslip. The MSgg agar is to the bottom and condensation is visible on the glass bottom on top. Cross sections of wrinkles are visible. **(C)** Confocal image of the biofilm cross section showing wrinkles and the σ^B expression gradient. Scale bar is 100 µm.

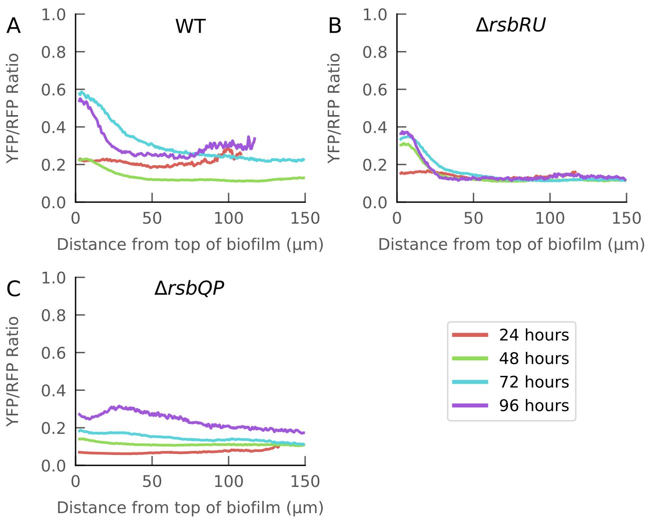

Supplementary Fig. 2. P$_{sigB}$-YFP gradient progression over time for WT, Δ*rsbRU*, and Δ*rsbQP* backgrounds. (A-C) Mean gradients for each genetic background over time for the P$_{sigB}$-YFP signal divided by the constitutively expressed P$_{sigA}$-RFP. (A) WT 24 hours: n = 11, 2 experiments. 48 hours: n = 24 from 5 experiments. 72 hours: n = 18, 4 experiments. 96 hours: n = 6 from 2 experiments. (B) Δ$rsbRU$ 24 hours: n = 6 from 1 experiment. 48 hours: n = 18 from 3 experiments. 72 hours: n = 37 from 4 experiments. 96 hours: n = 4 from 1 experiment. (C) Δ$rsbQP$ 24 hours: n = 7 from 1 experiment. 48 hours: n = 16 from 3 experiments. 72 hours: n = 13, 3 experiments. 96 hours: n = 4 from 1 experiment. Source data are provided as a Source Data file.

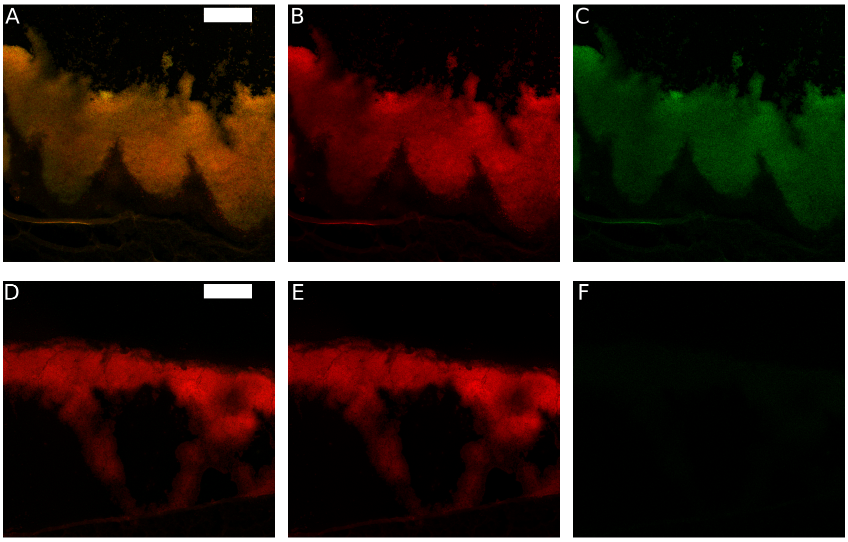

Supplementary Fig. 3. σ^W driven YFP expression is distributed uniformly across biofilm colonies. **(A-C)** *B. subtilis* strain with P_{ydbS}-YFP (green) and P_{sigA}-RFP (red, constitutive) reporters at 48 hours. **(A)** merged channels, **(B)** red channel, **(C)** green channel. **(D-F)** A strain with only the P_{sigA}-RFP (constitutive) fluorescent reporter. **(D)** merged channels, **(E)** red channel, **(F)** green channel. For both images, acquisition settings and fluorescence channel intensity scaling are the same. Scale bar is 100 µm.

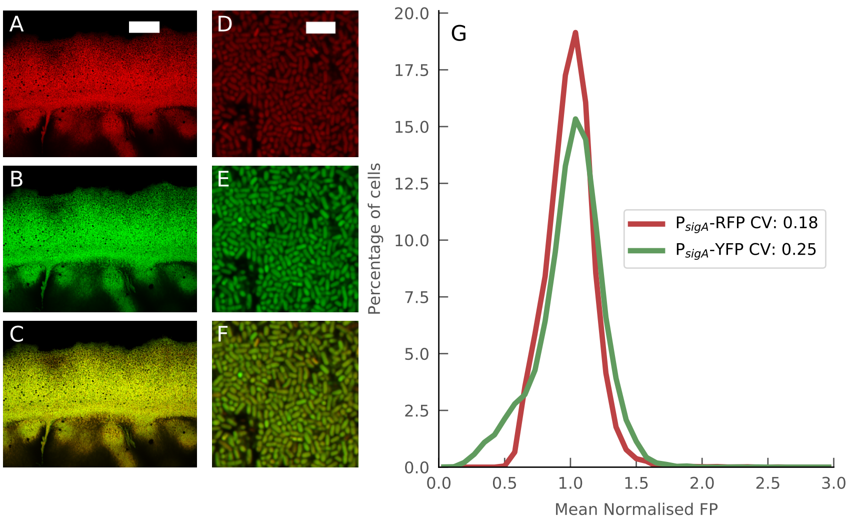

Supplementary Fig. 4. $\mathbf{P}_{sigA}$-YFP shows similar expression profile to $\mathbf{P}_{sigA}$-RFP. **(A-C)** P_{sigA}-YFP is expressed uniformly across the biofilm. Scale bar is 100 µm. **(A)** P_{sigA}-RFP red channel only, **(B)** P_{sigA}-YFP green channel and **(C)** a composite image of a strain containing P_{sigA}-YFP and P_{sigA}-RFP reporters. No gradient in expression in either reporter is observed. **(D-F)** P_{sigA}-YFP is expressed homogeneously in single cells. Scale bar is 5 µm. **(D)** red channel only, **(E)** green channel and **(F)** a composite high magnification image of the same strain shows uniform expression.
(G) P_{sigA}-YFP (green plot) expression shows a similar expression distribution as P_{sigA}-RFP (red plot) in single cells. Histogram data is filtered to remove out of focus cells (see Materials and Methods). RFP (respectively YFP) values for each image are normalised by the mean RFP (YFP) signal of in-focus cells in that image. Data are 11795 cells, n = 3 from 1 experiment. Source data are provided as a Source Data file.

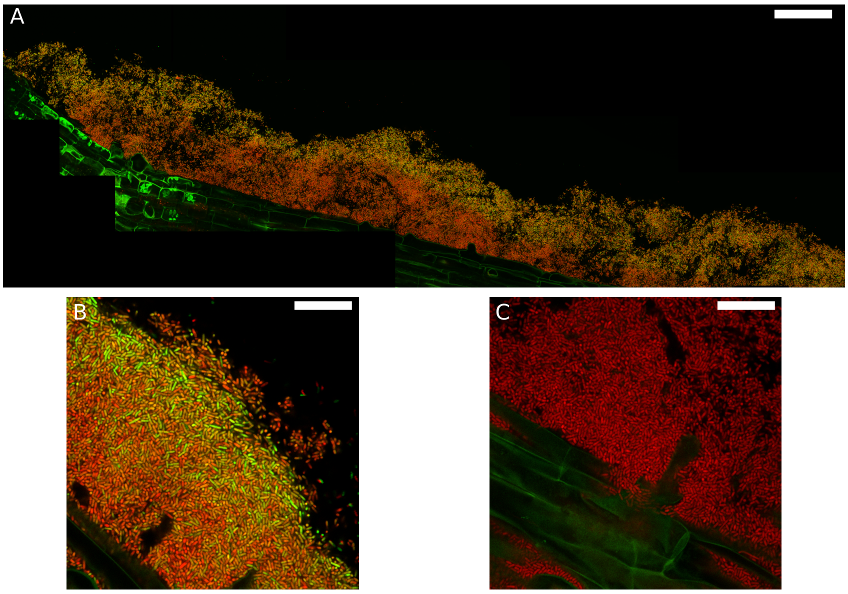

Supplementary Fig. 5. ***B. subtilis*** **forms biofilms on** ***A. thaliana*** **roots with distinct gradient of σ^B expression. (A)** Tile scan image of *B. subtilis* cells with P_{sigA}-RFP (constitutive, red) and P_{sigB}-YFP (green) fluorescent reporters. Scale bar is 100 µm. **(B)** High magnification images of the same strain as in (A), scale bar is 20 µm. **(C)** *B. subtilis* $\Delta\sigma^B$ strain with P_{sigA}-RFP and P_{sigB}-YFP reporters. Green signal on black is root autofluorescence. Scale bar is 20 µm.

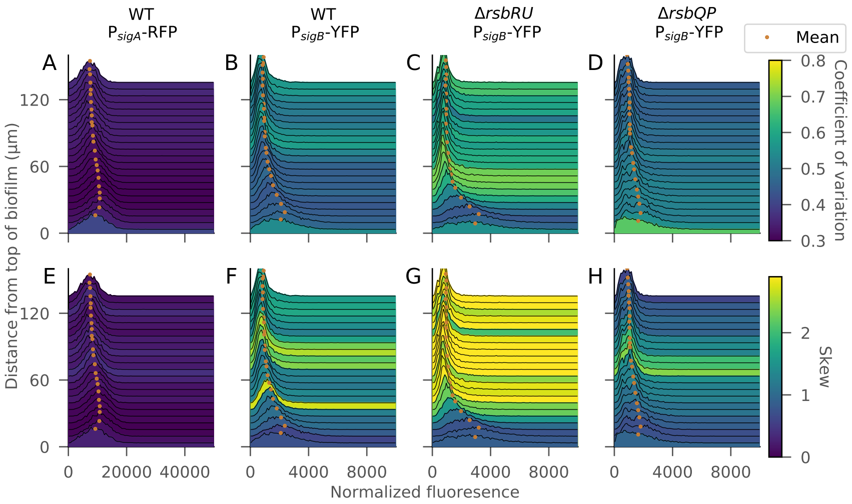

Supplementary Fig. 6. Characterisation of $\mathbf{P}_{sigB}$-YFP and $\mathbf{P}_{sigA}$-RFP single cell distributions through the biofilm (A–H) show the distribution of fluorescent reporter in bins 1 µm wide at depths every 6 µm from the top of the biofilm. The heights of the histogram curves are normalised by the number of cells in that bin while the histogram curves are coloured (A–D) according to their coefficient of variation and (E–H) by their skew. The orange dots mark the mean of the distribution. (A, E) show the distribution of a constitutive promoter P_{sigA}-RFP while all others show P_{sigB}-YFP for different σ^B backgrounds. The fluorescent values for each image are normalised by the mean cell fluorescence value of that image. (A, B, E, F) WT data from 359860 cells, n = 24 from 5 experiments. (B, G) $\Delta rsbRU$ data from 293357 cells, n = 18 from 3 experiments. (C, H) $\Delta rsbQP$ data from 232778 cells, n = 16 from 3 experiments. Source data are provided as a Source Data file.

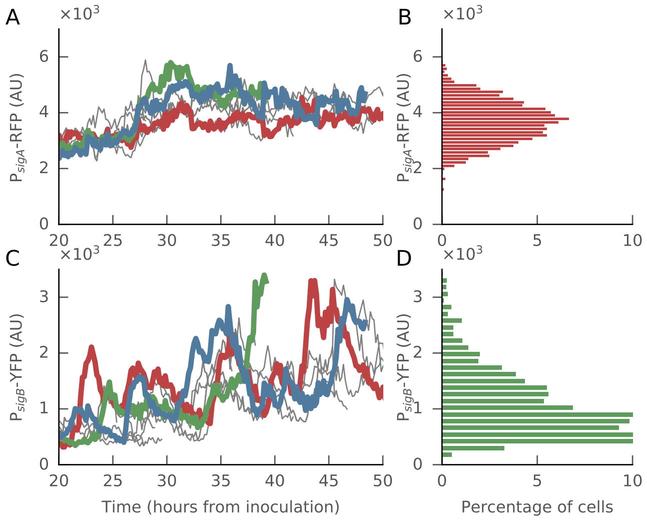

Supplementary Fig. 7. P_{sigB}-YFP levels display sustained pulses of expression in individual cells that are not apparent in the P_{sigA}-RFP control during biofilm growth. **(A)** Single cell lineages of P_{sigA}-RFP do not display significant fluctuations during biofilm growth. **(B)** Histograms of mean single cell P_{sigA}-RFP levels, CV = 0.19. **(C)** Single cell lineages of P_{sigB}-YFP display pulses of expression during biofilm growth. **(D)** Histograms of mean single cell P_{sigB}-YFP levels, CV = 0.55. The YFP/RFP ratio of this data is displayed in Fig. 4D. Traces are representative traces chosen from 67 cells tracked over 250 frames. Histograms n=1814 representing the 67 cells over 250 frames. Source data are provided as a Source Data file.

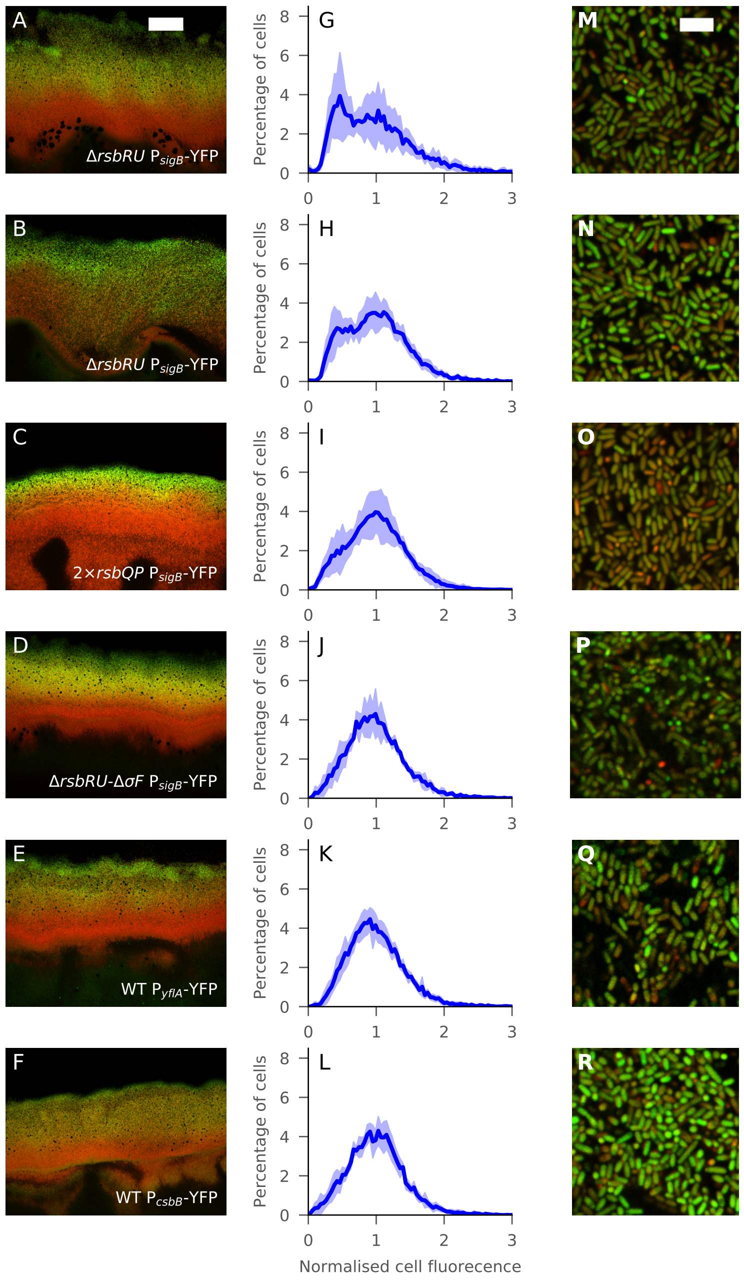

Supplementary Fig. 8. The σ^B gradient and the heterogeneous pattern of its activation does not require sporulation and is observed for multiple reporters of σ^B activity. A-F: representative images for each genotype. Green channels are scaled from 0 to 7 times the mean of the green channel. Scale bar is 100 µm. **G-L**: histograms of YFP expression normalised by the mean YFP fluorescence in each image. **M-R**: representative images showing variation in σ^B expression between neighbouring cells. Each green channel is scaled from 0 to 8 times the mean of the green channel. Scale bar is 5 µm. **(A, G, M)** Δ*rsbRU* (JLB088), **(G)** 12895 cells, n = 8, 2 experiments. **(B, H, N)** Δ*rsbRU* (NEB008), **(H)** 13287 cells, n = 6, 3 experiments. **(C, I, O)** 2×*rsbQP* (NEB011), **(I)** 24200 cells, n = 7, 3 experiments. **(D, J, P)** Δ*rsbRU* $\Delta\sigma^F$ (NEB018), **(J)** 11618 cells, n = 4, 2 experiments. **(E, K, O)** WT-P_{yflA}-YFP (NEB024), **(K)** 17383 cells, n = 7, 3 experiments. **(F, L, R)** WT-P_{csbB}-YFP (NEB026), **(L)** 10276 cells, n = 4, 2 experiments. Source data are provided as a Source Data file.

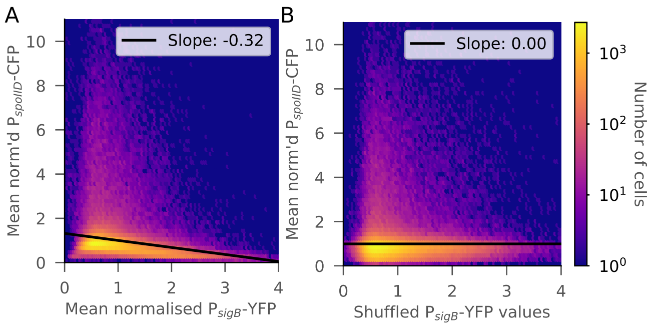

Supplementary Fig. 9. Expression of P_{sigB}-YFP and P_{spoIID}-CFP are negatively correlated in *B. subtilis* biofilms. (A) Bivariate histogram showing the frequency of cells co-expressing P_{sigB}-YFP and P_{spoIID}-CFP. The black line is the linear regression of the data highlighting the negative slope. The fluorescent values are mean normalised for each image. The data is the same as that plotted in Figure 7C, and represent two large tilescan images (each covering about 3 mm of biofilm) from two different experiments and 372689 cells. (B) shows exactly the same data as (A) but the P_{sigB}-YFP values have been randomly assigned to other cells. The linear regression now has slope 0. Source data are provided as a Source Data file.

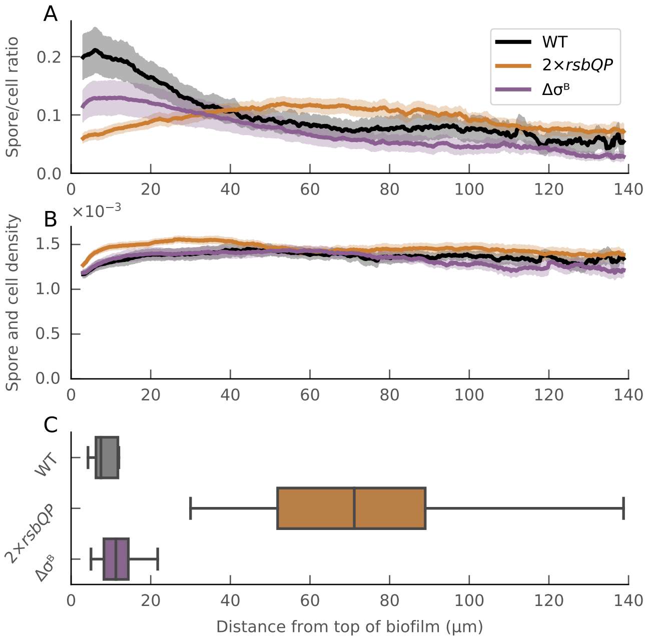

Supplementary Fig. 10. Sporulation peaks at the top of the biofilm in WT and σ^B deletion backgrounds, but not in 2×*rsbQP* strain. **(A)** shows the mean spore density for WT (black), σ^B deletion (purple), 2×*rsbQP* (orange) backgrounds. For WT and $\Delta\sigma^B$ n = 11 from 4 experiments, for 2×*rsbQP* n = 12 from 4 experiments. The graph represents the ratio of the amount of cells identified as spores (by expression of the P_{sspB}-YFP fluorescent marker) to the combined amount of all cells expressing P_{sigA}-RFP and spores (See Materials and methods for details of quantification). Error bars show the SEM. **(B)** shows the density of cells (spore or non-spore) for the same three strains (see Materials and methods for details of quantification). Error bars show the SEM. **(C)** Boxplots showing the distribution of the locations of the gradient peak of the spore/cell ratio (smoothed by mean rolling window) for each strain in the dataset. The box shows the median and quartiles of the dataset while the whiskers extend to show the extent of the distribution. Source data are provided as a Source Data file.

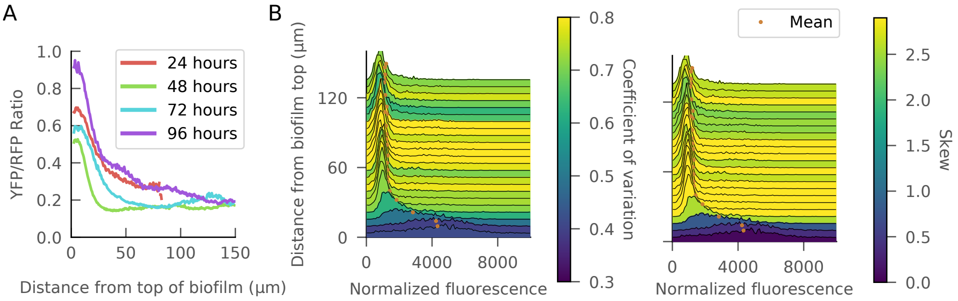

Supplementary Fig. 11. P_{sigB}-YFP gradient progression over time for the 2×*rsbQP* strain containing two copies of *rsbP* and *rsbQ*. (A) Mean gradients over time for the P_{sigB}-YFP signal divided by the constitutively expressed P_{sigA}-RFP. **(B)** shows how the distribution of P_{sigB}-YFP signal varies as we move away from the top of the biofilm at 48 hours. The histograms are coloured by their Coefficient of Variation and Skewness respectively. Values are in bins 1 µm wide at depths every 6 µm from the top of the biofilm. The height of the histogram curves are all normalised by the number of cells in that bin. The orange dots mark the mean of the distribution. Data at 24 hours: n = 5, 1 experiment. 48 hours: n = 7, 1 experiment. 72 hours: n = 8, 2 experiments. 96 hours n = 5, 1 experiment. Source data are provided as a Source Data file.

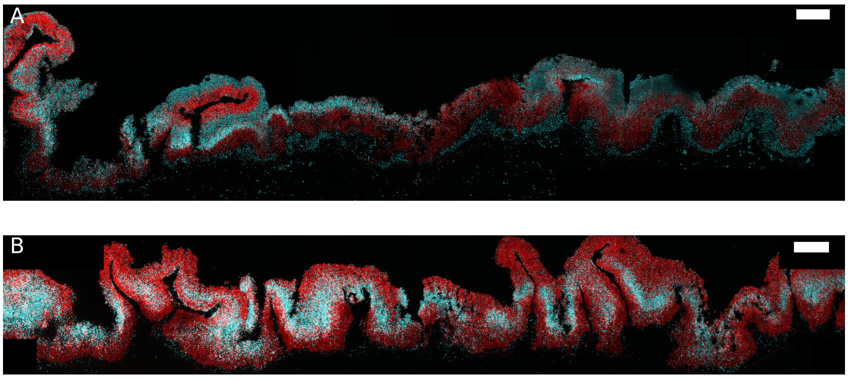

Supplementary Fig. 12. Representative biofilm cryoslices showing spore distribution. These images show the full context for the zoomed in sections in Fig. 7. Both images show *B. subtilis* cells with P_{sigA}-RFP (constitutive, red) and P_{sspB}-CFP (cyan) fluorescent reporters. **(A)** Shows the WT and **(B)** the 2×*rsbQP* strain. Scale bars are 100 µm.

Supplementary References

1. J. C. W. Locke, J. W. Young, M. Fontes, M. J. H. Jiménez, and M. B. Elowitz. Stochastic pulse regulation in bacterial stress response. *Science*, 334(6054):366–369, 2011. DOI: 10.1126/science.1208144.

2. A. Eldar, V. K. Chary, P. Xenopoulos, M. E. Fontes, O. C. Loson, J. Dworkin, P. J. Piggot, and M. B. Elowitz. Partial penetrance facilitates developmental evolution in bacteria. *Nature*, 460(7254):510–514, 2009. ISSN 0028-0836. DOI: 10.1038/nature08150.

3. H. Vlamakis, C. Aguilar, R. Losick, and R. Kolter. Control of cell fate by the formation of an architecturally complex bacterial community. *Genes Dev.*, 22(7):945–953, 2008. ISSN 08909369. DOI: 10.1101/gad.1645008.

4. J. Park, M. Dies, Y. Lin, S. Hormoz, S. E. Smith-Unna, S. Quinodoz, M. J. Hernández-Jiménez, J. Garcia-Ojalvo, J. C. W. Locke, and M. B. Elowitz. Molecular time sharing through dynamic pulsing in single cells. *Cell Systems*, 6(2):216–229.e15, 2018. ISSN 2405-4712. DOI: 10.1016/j.cels.2018.01.011.

5. M. A. Konkol, K. M. Blair, and D. B. Kearns. Plasmid-encoded ComI inhibits competence in the ancestral 3610 strain of *Bacillus subtilis*. *J. Bacteriol.*, 195(18):4085–4093, 2013. ISSN 00219193. DOI: 10.1128/JB.00696-13.

6. B.-M. Koo, G. Kritikos, J. D. Farelli, H. Todor, K. Tong, H. Kimsey, I. Wapinski, M. Galardini, A. Cabal, J. M. Peters, A.-B. Hachmann, D. Z. Rudner, K. N. Allen, A. Typas, and C. A. Gross. Construction and analysis of two genome-scale deletion libraries for bacillus subtilis. *Cell systems*, 4: 291–305.e7, 2017. ISSN 2405-4712. DOI: 10.1016/j.cels.2016.12.013.
