# Supplementary material for: Stochastic pulsing of gene expression enables the generation of spatial patterns in Bacillus subtilis biofilms
Source: Nat Commun. 2020 Feb 19;11:950. doi: 10.1038/s41467-020-14431-9 (PMC7031267; doi:10.1038/s41467-020-14431-9)
Supplement: Supplementary file 3 — Description of Additional Supplementary Files [file 41467_2020_14431_MOESM3_ESM.pdf]

Description of Additional Supplementary Files

File Name: Supplementary Movie 1
Description: A movie of *ΔrsbRU* biofilm formation showing the development of the σB expression gradient and wrinkle formation. PsigA-RFP in red, PsigB-YFP in green. The time is in hours and minutes from inoculation.

File Name: Supplementary Movie 2
Description: A movie of *ΔrsbRU* biofilm formation tracking a single cell showing the expression of PsigA-RFP (red line) and PsigB-YFP (green line) and demonstrating pulsatile PsigB-YFP expression.

File Name: Supplementary Movie 3
Description: A movie of *ΔrsbRU* 2×rsbQP biofilm formation showing single cells expressing PsigA-RFP (red) and PsigB-YFP (green) and demonstrating PsigB-YFP pulsatile expression. The time is in hours and minutes from inoculation.
